# Systemic Factors Related to Intraocular Levels of Interleukin-6 and Vascular Endothelial Growth Factor in Diabetic Retinopathy

**DOI:** 10.1155/2019/4831967

**Published:** 2019-07-17

**Authors:** Byung Ju Jung, Mee Yon Lee, Sohee Jeon

**Affiliations:** ^1^Apgujung St. Mary's Eye Center, Seoul, Republic of Korea; ^2^Department of Ophthalmology, Uijeongbu St. Mary's Hospital, College of Medicine, The Catholic University of Korea, Uijeongbu, Republic of Korea; ^3^Keye Eye Center, Seoul, Republic of Korea

## Abstract

This study is for identifying systemic factors correlating with intraocular levels of interleukin-6 (IL-6) and vascular endothelial growth factor (VEGF) in diabetic retinopathy. Forty-two consecutive patients undergoing pars plana vitrectomy (PPV) for PDR were included in this cross-sectional study. The aqueous humor was sampled just prior to PPV for assay of IL-6 and VEGF. One day before PPV, patient characteristics were recorded and a number of systemic markers were amassed, including fasting and postprandial glucose, homeostasis model assessment- (HOMA-) IR, HOMA-beta, C-peptide, insulin, total cholesterol, triglycerides, high-density lipoprotein cholesterol, low-density lipoprotein cholesterol, apolipoprotein- (Apo-) A, Apo-B, and lipoprotein A (Lp-A). Relationships between systemic determinants and intraocular cytokine levels were analyzed by regression analysis. Mean levels of IL-6 and VEGF were 15.3 pg/mL (range, 2.4–10124.5 pg/mL) and 21.1 pg/mL (range, 3.2–766.1 pg/mL), respectively. After adjustment for age, gender, duration of diabetes, and BMI, multivariate analysis showed significant association of smoking (*p*=0.002) and HOMA-IR (*p*=0.003) with intraocular IL-6 levels, while intraocular VEGF and systemic Lp-A levels correlated significantly (*p*=0.032). Insulin resistance and smoking status impacted intraocular levels of IL-6, while intraocular VEGF levels were influenced by Lp-A. An appreciation for the relationship between systemic factors and intraocular cytokines may help elucidate the complex pathophysiology of diabetic retinopathy.

## 1. Introduction

Diabetic retinopathy (DR) is one of the most common microvascular complications of diabetes mellitus (DM) and is a leading cause of lost vision in developed countries [1]. Efforts to identify systemic factors contributory to DR and their respective mechanisms have been enormous. Consequently, a number of variables, such as hyperglycemia [2], hypertension [3], dyslipidemia [4], alcohol consumption [5], insulin resistance [6], obesity [7], and genetic factors [8–11], have been cited as risk factors for DR.

Recent progress in immunoassays, enabling use of exceedingly small aliquots, has greatly expanded the study of various intraocular cytokines. A complex process, both inflammatory and angiogenic in nature, is now implicated in the development of DR, to include intraocular upregulation of the following cytokines: vascular endothelial growth factor (VEGF) [12, 13], interleukin- (IL-) 1*β* [14], IL-2 [15], IL-6 [16, 17], IL-8 [18], transforming growth factor- (TGF-) *β* [19], tumor necrosis factor- (TNF-) *α* [14], interferon-induced protein- (IP-) 10 [20], and monocyte chemoattractant protein- (MCP-) 1 [21]. Through intricate pathways, these mediators are accountable for the neovascularization and breech of the blood-retinal barrier that occur. Among them, VEGF is key in the development and progression of DR, conferring conformational changes to the tight junctions of retinal vascular endothelial cells and acting in large part to increase vascular permeability and promote proliferation of vessels [22, 23]. IL-6, on the contrary, is a multifunctional cytokine with acute phase reactant and immunomodulatory roles [24]. An *in vitro* study of IL-6 describes rearrangement of actin filaments and morphologic changes of endothelial cells under its influence, leading to heightened endothelial permeability [25]. IL-6 also has the capacity to induce VEGF expression [26], further increasing vascular permeability. Intraocular IL-6 level reportedly parallels the severity of DR and diabetic macular edema [27].

We believe that the intraocular cytokine milieu is affected by various systemic factors, culminating in the development and progression of DR. However, there is limited support in this regard. The present study therefore focused on the relationships of intraocular cytokines (IL-6 and VEGF) with a host of relevant systemic parameters, including hyperglycemia, hypertension, dyslipidemia, smoking, alcohol consumption, insulin resistance, and obesity.

## 2. Materials and Methods

The study protocol incorporated tenets of the Declaration of Helsinki and was approved by the institutional ethics committee at St. Mary's Hospital of Seoul, South Korea, where all procedures and evaluations were conducted. Signed informed consent was obtained from each participant after detailed explanation of study objectives and design, including specific scientific investigations and adjunctive surgical procedures.

Exclusion criteria by history were as follows: (1) pharmacologic intervention or laser photocoagulation of eyes for study within the prior six months, (2) pharmacologic intervention of fellow eyes within the prior three months, (3) ocular disease other than DR, (4) prior ocular surgery of the study eye, (5) systemic inflammatory disease other than DM, and (6) any systemic disease with the potential to skew HbA1c results.

A standard questionnaire detailing medical history and lifestyle was administered to each enrollee by a trained physician. Time since diagnosis of DM, DM treatments received, age, and gender were self-reported. Alcohol intake at least once a week in the prior six months qualified as positive. We defined current smokers as people who reported smoking at least 100 cigarettes during their lifetime and who currently smoke every day or some days [28]. Blood pressure (BP) was taken in the sitting position from the right arm, reading to the nearest 2 mmHg with a mercury sphygmomanometer (A&D Company Ltd, Tokyo, Japan). The mean of two attempts, taken five minutes apart, served as the official reading. Hypertension was defined by cut points (systolic BP ≥ 130 mmHg; diastolic BP ≥ 85 mmHg) or based on use of antihypertensives. From anthropometric measurements, body mass index (BMI) was calculated as weight/height [2] and expressed in kilograms per square meter.

Blood samples were drawn between 7:00 AM and 9:00 AM one day before PPV for processing by the same laboratory, after a minimum fasting of eight hours. Glucose, total cholesterol (TC), triglyceride (TG), high-density lipoprotein cholesterol (HDL-C), low-density lipoprotein cholesterol (LDL-C), apolipoprotein-A (Apo-A), apolipoprotein-B (Apo-B), and lipoprotein A (Lp-A) were measured. HbA1c was monitored by high-performance liquid chromatography (HPLC; Bio-Rad Co, Hercules, CA), and serum insulin concentration was determined by RIA (Diagnostic Products Corp, Los Angeles, CA).

The homeostasis model assessment (HOMA) formula quantified insulin resistance as follows [29]: [(HOMA-IR (mg/dL *∗* mIU/mL) = fasting glucose (mg/dL) *∗* fasting insulin (mIU/mL)/405].

Similarly, *β*-cell function was gauged by the following HOMA-*β* formula: [HOMA-*β*(%) = fasting insulin (mIU/ml) *∗* 360/(fasting glucose (mg/dL) − 63)].

Early morning spot urine samples were screened for microalbuminuria via particle-enhanced turbidimetric inhibition assay, with 24 h albumin excretion calculated from results.

Each patient was draped, and a lid speculum was inserted. Povidone-iodine 5% was then instilled into the conjunctival sac and lid margin, and undiluted aqueous humor (50–100 *μ*L) was harvested from the anterior eye chamber under retrobulbar anesthesia (just prior to pars plana vitrectomy). Samples were stored at −70°C for later analysis. One surgeon performed all procedures (SJ).

Suspension array technology (xMAP; Luminex Corp, Austin, Texas, USA) was engaged for analysis of the aqueous humor, using capture bead kits (Beadlyte; Upstate Biotechnology, Lake Placid, NY) for IL-6 and VEGF detection. Samples were incubated overnight, and testing was conducted according to manufacturer's instructions [27]. Standard curves for each cytokine were generated in duplicate utilizing the kit-supplied reference cytokine concentrations. To protect the beads from light, all incubations were performed in the dark at room temperature.

Results were expressed as mean values ± SD (continuous variables) or as percentages (categorical variables). IL-6, VEGF, insulin, fasting glucose, HOMA-IR, HOMA-beta, TC, TG, HDL-C, LDL-C, Apo-B, and Lp-A values were log-transformed since none displayed normal distribution by the Shapiro–Wilk test. Respective relationships of IL-6 and VEGF levels with various systemic markers—age, gender, duration of diabetes, BMI, systolic and diastolic blood pressure, alcohol, smoking, hyperglycemia (fasting and postprandial glucose), insulin resistance (HOMA-IR, HOMA-beta, C-peptide, and insulin), and dyslipidemia (TG, HDL-C, LDL-C, Apo-A, Apo- B, and Lp-A)—were subjected to univariate regression. The Spearman rank correlation coefficient was determined to assess the association between continuous variables since none displayed normal distribution by the Shapiro–Wilk test. Independent variables significantly associated with IL-6 or VEGF levels in univariate analysis (*p* < 0.05) and potentially confounding parameters were included as independent covariables in multivariate analysis by multiple regression analysis. The Mann–Whitney test was used to address the difference of IL-6 between smokers and nonsmokers. All computations were done using SAS version 9.1 (SAS Institute Inc, Cary, NC) or MedCalc version 11.2.1.0 (MedCalc Software bvba, Mariakerke, Belgium). Statistical significance was set at *p* < 0.05.

## 3. Results

A total of 42 consecutive patients (42 eyes) with DR were examined in this cross-sectional study. Clinical and biochemical characteristics of the group are shown in Table 1. Male patients predominated (73.8%), with a mean age of 56.0 years (range, 19.0–71.0 yrs). Forty-one patients suffered from type 2 diabetes, while one patient suffered from type 1 diabetes (mean duration, 10.5 yrs; range, 1.0–37.0 yrs). Thirty-five eyes showed high-risk PDR (ETDRS level 75, 75), and seven eyes showed advanced PDR (ETDRS level 81, 85). Twenty-nine eyes (69.0%) had a history of laser photocoagulation.

**Figure 1:**
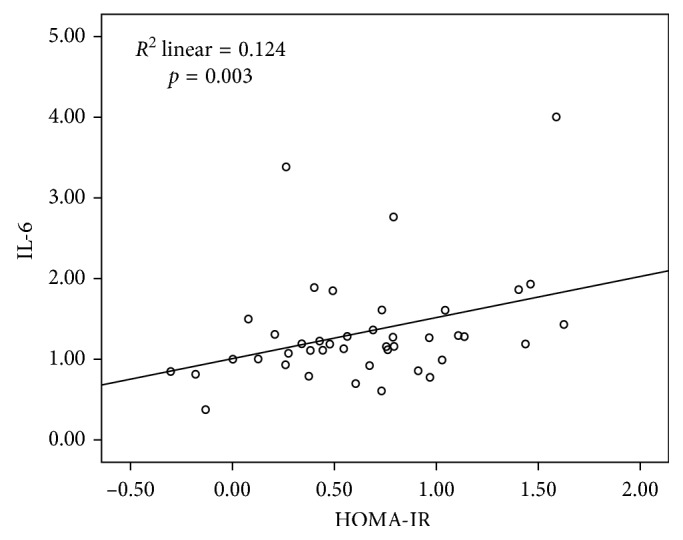
Correlation of the intraocular IL-6 level with HOMA-IR.

Median IL-6 level was 15.3 pg/mL (range, 2.4–10124.5 pg/mL). Simple regression analysis showed significant correlation of intraocular IL-6 levels with HOMA-IR (*r* = 0.353, *p*=0.022) and smoking status (*r* = 0.135, *p*=0.017). While fasting glucose and HbA1c showed positive trends, statistical significance was lacking (*p*=0.076 and *p*=0.077, respectively) (Table 2). When HOMA-IR and smoking status, as significant correlates, were incorporated into the multiple regression model, both were confirmed as independent and significant correlates of intraocular IL-6 levels, after adjustment for age, gender, duration of diabetes, and BMI (Table 2; Figure 1).

The median IL-6 level was 15.3 pg/mL (range, 2.4–579.4 pg/mL) in nonsmokers, while it was 20.1 pg/mL (range, 9.9–10124.5 pg/mL) in smokers. There was no significant difference in IL-6 levels between smokers and nonsmokers (*p*=0.275, Mann–Whitney test).

The median VEGF level was 21.1 pg/mL (range, 3.2–766.1 pg/mL), with lipoprotein A as the sole parameter to correlate significantly with intraocular VEGF levels before (*r* = 0.160, *p*=0.014) and after adjustment for age, gender, duration of diabetes, and BMI (*p*=0.032) (Table 3). A positive trend noted for smokers fell short of statistical significance (*p*=0.079).

## 4. Discussion

The present investigation suggests a significant link between intraocular IL-6 levels and two systemic factors—insulin resistance (represented as HOMA-IR) and smoking. To the best of our knowledge, this is the first study to demonstrate such a relationship. This link may aid our understanding of DR pathogenesis and the retinal microvascular complications thereof.

Insulin resistance in effect equates with a cluster of risk factors, namely, resistance to insulin-stimulated glucose uptake, glucose intolerance, and hyperinsulinemia [30]. More importantly, it has been increasingly recognized that insulin resistance has a major hand in inflammatory pathways [31], as well as in various aspects of macrovascular [6, 32] and microvascular disease [6]. In the setting of insulin resistance, systemic cytokine elevations (including IL-6) have been implicated in acute-phase response (C-reactive protein) induction, heightened endothelial production of vascular cell adhesion molecule 1 (VCAM-1) and monocyte chemotactic protein 1 (MCP-1), and consequently the development of macrovascular disease [33]. Our study corroborates further by establishing a significant correlation between insulin resistance and intraocular IL-6—a cytokine so crucial for initiation and progression of DR [16, 17]. Although far from conclusive, this link may explain why patients with DR often suffer progressive visual loss despite relatively good glycemic control. In any event, the relationship between insulin resistance and various clinical manifestations of DR merits continued research.

Interestingly, our study also revealed a significant relationship between smoking status and intraocular levels of IL-6 in DR, and this association did not diminish after adjustment for insulin resistance. Moreover, the intraocular VEGF level did trend higher with smoking as well. Smoking has been proven as a significant risk factor in various ocular disease states, such as age-related macular degeneration, cataracts, thyrotoxic ophthalmopathy, uveitis, and diabetic retinopathy [34]. Present results imply that smoking may provoke ocular disease by increasing intraocular levels of inflammatory and angiogenic cytokines. Smoking and systemic IL-6 levels have been linked in one study by Reichert et al. [35] although intraocular levels were not specifically addressed. However, we found no significant difference in IL-6 levels between smokers and nonsmokers. We speculate that a small number of smokers enrolled in the present study, nonnormality of distribution in IL-6, and subject nature of definition of smoking might affect the outcomes. A deeper look into the impact of smoking on various intraocular cytokines is thus warranted.

Lp-A is an altered form of LDL, incorporating the apolipoprotein-B-100 moiety of LDL. Elevated Lp-A is associated with a higher risk of coronary and cerebrovascular disease, independent of total cholesterol or LDL levels [36]. Owing to structural similarities between Lp-A plasminogen and tissue plasminogen activator, Lp-A competes with plasminogen for its binding site, leading to reduced fibrinolysis [36]. Previous studies have identified elevated concentrations of the Lp-A level as an independent risk factor for progression of DR in type 2 diabetes and for development of retinal vein occlusion [37, 38]. We therefore speculate that the atherogenic effect of Lp-A may aggravate retinal capillary blockage to increase intraocular levels of VEGF and consequently promote the progression of DR.

At present, consensus roughly upholds that hyperglycemia and intraocular VEGF are contributory to DR. However, no significant relationship between hyperglycemia and intraocular levels of IL-6 or VEGF has emerged here or from another prior investigation [13]. On the contrary, we do not consider such an association unreasonable [39]. One particular study has shown hyperglycemia to stimulate the synthesis and production of IL-6 by human peripheral blood monocytes *in vitro* [39]. We thus maintain that hyperglycemia of chronic nature, rather than the glycemic status (i.e., fasting glucose and HbA1c levels), is largely responsible for the development and progression of DR. It may well be that a better biomarker for chronic hyperglycemia is needed to decisively link DR with hyperglycemia and intraocular cytokine activity.

We speculate that the subject and ambiguous nature of definition of smoking may influence the results. And our study has limitations, such as a small sample size and the imbalance between the sexes. Further studies with larger sample sizes are warranted to confirm our preliminary results.

Although the impact of systemic factors on DR has been the focus of extensive research, some outcomes have been inconsistent, perhaps due to the highly complex mechanisms that are involved. Despite conflicting evidence, we view such pursuits as fully warranted and capable of adding substantially to our understanding of DR.

## 5. Conclusions

This study showed intraocular IL-6 levels were associated with insulin resistance and smoking status, while intraocular VEGF levels were influenced by Lp-A. An appreciation for the relationship between systemic factors and intraocular cytokines may help elucidate the complex pathophysiology of DR.

## Figures and Tables

**Table 1:** Clinical and biochemical characteristics of study population (*n* = 42).

	Eyes
Gender, male	31 (73.8)
Age, years	56.0 (19.0–71.0)
Duration of diabetes (years)	10.5 (1.0–37.0)
Body mass index (kg/m^2^)	23.9 (20.0–31.6)
History of laser photocoagulation, yes	29 (69.0)
Tractional retinal detachment, yes	13 (31.0)
Epiretinal membrane, yes	4 (9.5)
Vitreous hemorrhage, yes	25 (59.5)
Insulin treatment, yes	27 (45.0)
Hypertension, yes^*∗*^	28 (66.7)
Systolic blood pressure (mmHg)	131.5 (98.0–170.0)
Diastolic blood pressure (mmHg)	80.0 (53.0–110.0)
Alcohol consumption, yes^†^	8 (19.1)
Smoking status, yes^‡^	8 (19.1)
HbA1c (%)	8.2 (5.7–13.3)
Fasting glucose (mg/dL)	149.0 (70.0–302.0)
Postprandial glucose (mg/dL)	205.0 (85.0–405.0)
HOMA-IR	4.4 (0.5–42.4)
HOMA-beta	58.3 (5.8–1827.3)
C-peptide (ng/ml)	2.1 (0.0–6.2)
Insulin (*μ*U/ml)	11.6 (1.6–81.7)
Microalbuminuria (*μ*g/ml)	86.1 (3–2400.7)
Serum BUN (mg/dL)	66.1 (1.4–331.3)
Serum creatinine (mg/dL)	0.9 (0.4–11.9)
Total cholesterol (mg/dL)	177.5 (92.0–431.0)
Triglyceride (mg/dL)	128.5 (30.0–492.0)
HDL cholesterol (mg/dL)	38.5 (23.0–84.0)
LDL cholesterol (mg/dL)	93.5 (39.0–249.0)
Apolipoprotein-A (mg/dL)	118.0 (72.0–195.0)
Apolipoprotein-B (mg/dL)	87.0 (43.0–203.0)
Lipoprotein A (mg/dL)	15.2 (2.6–150.0)

^*∗*^Hypertension was defined using the average of two blood pressure readings with cut points of systolic blood pressure ≥140 mmHg, diastolic blood pressure ≥90 mmHg, or hypertension medication use. ^†^Alcohol drinking status was described as yes if the patient consumed alcohol at least once a week in the past 6 months. ^‡^Smoking status was determined by seven cigarettes per week in the past 6 months. HbA1c, glycated hemoglobin; HOMA-IR, homeostasis model assessment-insulin resistance; HOMA-beta, homeostasis model assessment-beta; BUN, blood urea nitrogen; HDL, high-density lipoprotein; LDL, low-density lipoprotein; IL, interleukin; VEGF, vascular endothelial growth factor. Values are represented in frequency and percentage for categorical variables and mean and range for continuous variables.

**Table 2:** Correlation of the intraocular IL-6 level with systemic factors.

	Simple Linear regression		Multiple linear regression
	Regression coefficient	*p* value	*p* value
Gender	0.002	0.786	
Age	0.008	0.560	0.225
Duration of diabetes	0.021	0.357	0.512
History of laser photocoagulation	0.016	0.448	
Body mass index	0.002	0.759	0.436
Systolic BP	0.002	0.751	
Diastolic BP	0.022	0.343	
Hypertension	0.008	0.580	
Alcohol consumption	0.001	0.826	
Smoking status	0.135	0.017	0.002
Insulin	0.027	0.295	
HbA1C	0.076	0.077	
Fasting glucose	0.077	0.076	
HOMA-IR	0.456	0.002	0.002
HOMA-beta	0.008	0.574	
C-peptide	0.027	0.296	
Total cholesterol	0.016	0.430	
Triglycerides	0.055	0.134	
HDL cholesterol	0.017	0.410	
LDL cholesterol	0.023	0.337	
Apolipoprotein-A	0.001	0.886	
Apolipoprotein-B	0.001	0.830	
Lipoprotein A	0.002	0.806	

BP, blood pressure; HbA1c, glycated hemoglobin; HOMA-IR, homeostasis model assessment-insulin resistance; HOMA-beta, homeostasis model assessment-beta; BUN, blood urea nitrogen; HDL, high-density lipoprotein; LDL, low-density lipoprotein.

**Table 3:** Correlation of the intraocular VEGF level with systemic factors.

	Simple linear regression		Multiple linear regression
	Regression coefficient	*p* value	*p* value
Gender	0.028	0.293	
Age	0.006	0.618	0.975
Duration of diabetes	0.021	0.364	0.552
History of laser photocoagulation	0.022	0.334	
Body mass index	0.014	0.456	0.532
Systolic BP	0.001	0.812	
Diastolic BP	0.005	0.667	
Hypertension	0.012	0.491	
Alcohol consumption	0.069	0.091	
Smoking status	0.075	0.079	
Insulin	0.006	0.614	
HbA1C	0.024	0.325	
Fasting glucose	0.005	0.667	
HOMA-IR	0.0004	0.894	
HOMA-beta	0.004	0.690	
C-peptide	0.042	0.192	
Total cholesterol	0.004	0.682	
Triglycerides	0.024	0.331	
HDL cholesterol	0.043	0.186	
LDL cholesterol	0.005	0.641	
Apolipoprotein-A	0.058	0.152	
Apolipoprotein-B	0.005	0.685	
Lipoprotein A	0.160	0.014	0.032

BP, blood pressure; HbA1c, glycated hemoglobin; HOMA-IR, homeostasis model assessment-insulin resistance; HOMA-beta, homeostasis model assessment-beta; BUN, blood urea nitrogen; HDL, high-density lipoprotein; LDL, low-density lipoprotein.

## Data Availability

The data used to support the findings of this study are available from the corresponding author upon request.
